# *Nicotiana tabacum*: From a Beneficial Plant to Harmful Products—A Comprehensive Review of Smokeless Tobacco and Its Adverse Health Effects

**DOI:** 10.1155/tswj/3369964

**Published:** 2026-04-24

**Authors:** Abir Chami, Afaf Allaoui, Hassane Mekhfi, Younes Zaid

**Affiliations:** ^1^ Materials, Nanotechnologies and Environment Laboratory, Department of Biology, Faculty of Sciences, Mohammed V University, Rabat, Morocco, um5.ac.ma; ^2^ Laboratory of Bio-Resources, Biotechnology, Ethnopharmacology and Health, Department of Biology, Faculty of Sciences, Mohammed First University, Oujda, Morocco; ^3^ Immunology and Biodiversity Laboratory, Department of Biology, Ain Chock Faculty of Sciences, Hassan II University, Casablanca, Morocco, uh2c.ac.ma

**Keywords:** *Nicotiana tabacum*, secondary effects, smokeless tobacco

## Abstract

*Nicotiana tabacum* is a cash crop that originated in America and was introduced to the world by Christopher Colombus. It is now cultivated in many countries with major producers including China, the USA, India, and Brazil. The plant was widely used by the indigenous people of America for different reasons (spiritual, hallucination, and medical). The medicinal usage of the plant was confirmed by many phytochemical and pharmacological studies on different parts of the plant. The plant essentially contains alkaloids (nicotine is the major component) and phenolic compounds such as flavonoids, tannins, and many phenolic acids. Glycosides and terpenes are also present in *N. tabacum*. These components are responsible for many pharmacological effects. Besides the therapeutic usage of the plant, the leaves of the plant served as a hallucination agent; they were either smoked or chewed. Nowadays tobacco products are universally used. Smokeless tobacco products are numerous and diversified; they can be handmade or manufactured. Unlike the plant, smokeless tobacco products are harmful to health. Many products exist around the world with different compositions and names, such as paan, zarda, toombak, khaini, naswar, loose leaf, moist snuff, snus, and shammah. These products have been confirmed to cause many serious pathological conditions. In addition to being addictive, they can lead to various types of cancer especially oral cancer, hypertension, cardiovascular diseases, fertility issues, and fetal damage when consumed by pregnant women. They also increase the risk of thrombosis and so on. While the harmful effects of some products have been scientifically proven, others (e.g., chemma) have not. This highlights the importance of scientific investigation to confirm their potential risks.

## 1. Introduction

*Nicotiana tabacum,* commonly known as tobacco, is a perennial plant that belongs to the Solanaceae family. The plant is native to tropical and subtropical America and is currently grown worldwide as a cash crop [1]. Introduced to the world after the discovery of America and named after Jean Nicot, French ambassador, who sent seeds of the plant to France [2]. The native Americans were probably the first people who used tobacco plant for many purposes [3]. Nowadays *N. tabacum* products are mainly consumed in the form of smoking, chewing, snuffing, or dipping as an addictive substance. There are many types of smokeless tobacco (SLT) products, and each country usually has its own types, which can be handmade or manufactured. The chemical composition of SLT in general contains a lot of harmful components, especially carcinogenic ones. In particular, recent reviews have comprehensively addressed the chemical composition and carcinogenic mechanisms of SLT products, including the formation of tobacco‐specific nitrosamines (TSNAs) during cultivation and curing and their mutagenic implications [4–6]. The main objective of this review is to describe the plant, explain its phytochemical composition and its pharmacological effects, and shed light on different types of SLT products and their harmful effects on human health. One of these products is chemma, a Moroccan product that was not studied before; one of our objectives is to introduce this product to the literature.

## 2. *Nicotiana tabacum*

### 2.1. Taxonomy of *Nicotiana tabacum*

**FIGURE 1:**
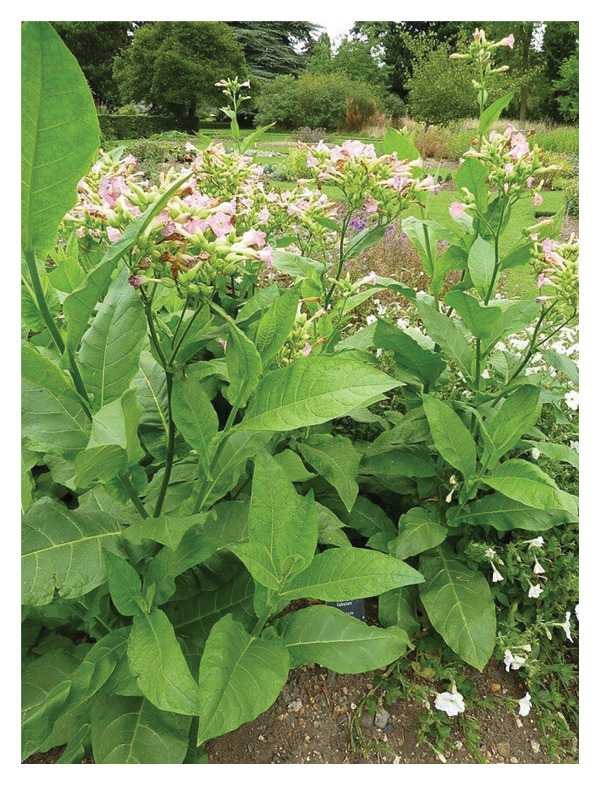
*Nicotiana tabacum* plants (image by Magnus Manske) [7].

*Nicotiana tabacum* (Figure 1) also known as the tobacco plant is a species that belongs to the genus *Nicotiana*, under the class of Magnoliopsida and the family of Solanaceae [8–10].

• Kingdom: Plantae [10]
• Phylum: Tracheophyta [10].
• Class: Magnoliopsida [10].
• Order: Solanales [10].
• Family: Solanaceae [10].
• Genus: *Nicotiana* [10].
• Species: *Nicotiana tabacum* L. [10].

### 2.2. Overview and Historical Background of the Plant

*N. tabacum* is an annual plant that grows to a height between 1 and 2 m with an erect stem with few branches [11].

The leaves are ovate (egg‐shaped), elliptic (oval‐shaped), or lanceolate (long and thin). They are normally sessile; however, they can occasionally have a petiole. They are green colored but can develop other colors during the curing process, which prepares them for use in tobacco products [12]. Leaves are the most valuable part of a plant. They are commercially harvested and processed into tobacco products [13]. The fruit of *Nicotiana* is a capsule, which is approximately 15–20 mm long and narrowly elliptic, ovoid, or orbicular. The flowers can be light red, light pink, or white, while the seeds are spherical or broadly elliptic, approximately 0.5 mm long, and brown with fluted ridges. The inflorescence is a panicle, a branching cluster of flowers. It has an identifiable rachis (central axis) and several compound branches [11].

Tobacco is an allotetraploid (2n = 4x = 48) species resulting from the hybridization of the diploid (2n = 12) ancestor species *Nicotiana sylvestris* and *Nicotiana tomentosiformis* about 200,000 years ago [14].

It is assumed to have originated in a region between North and South America. Evidence indicates that its cultivation took place at least 5000 years ago [15]. The indigenous people of America were the first users of the tobacco plant; they used to inhale the grounded plant through a hollow Y‐shaped piece of cane or pipe that was called a “tobago” or “tobaca” [3]. The word was later changed by the Spaniards to “tobacco” which is where the name of the plant comes from. They introduced it to Christopher Columbus in 1492 which was the beginning of the history of tobacco’s discovery [16]. In 1560, the French ambassador Jean Nicot introduced tobacco to the French court by sending the seeds obtained from Lisbon [17]. The name of *Nicotiana* likely originates in honor of the French ambassador [18]. Linnaeus gave the botanical name “*Nicotiana tabacum*” for the tobacco plant genus in 1753. In 1828, the active ingredient of tobacco was isolated and called *nicotine* [3].

### 2.3. Growth Requirements of *Nicotiana tabacum*

Currently, *N. tabacum* is cultivated in over 100 countries across approximately 4.2 million hectares of land, with major producers including China, the USA, India, and Brazil [19]. Like every plant, *N. tabacum* has its own specific requirements and environmental factors that influence its growth. It prefers warm climates with a frost‐free period of 100–130 days for optimal growth [20]. Shelfer mentioned that tobacco could grow in any climate but prefers and does best in tropical or semitropical areas [21]. Tobacco prefers well‐drained soil; poor drainage leads to standing water, causing root rot and stunting plant development [20]. It is generally grown in sandy and sandy‐loam soil [22]. The presence of adequate levels of essential nutrients is necessary for plant growth, but high nitrogen can lead to larger leaves with a lower quality for tobacco products [23]. The ideal soil pH can differ depending on various factors in the United States; the optimal pH range for tobacco cultivation is typically between 6.0 and 6.4. In contrast, in India, the preferred pH range is higher, ranging from 7.5 to 8.5. Meanwhile, in China, where high‐quality tobacco is cultivated, the soil pH in such areas generally falls between 5.0 and 7.0. Presently, within the academic sphere, there’s a consensus that the best pH range for achieving superior tobacco leaf production is approximately 5.5 to 6.5 [24].

## 3. Phytochemistry of *Nicotiana tabacum*

The tobacco plant is well‐known for its chemical diversity; more than 2500 compounds have been identified [25]. The phytochemical composition of *N. tabacum* may vary depending on factors such as processing conditions, temperature, maturation stage, time of harvesting, and storage. Many phytochemical studies on different varieties from different origins show that *N*. *tabacum* contains a lot of secondary metabolites. The following cited studies used fresh unprocessed plant materials (either leaves or stems) which were subsequently dried for analysis. Sandhya et al. have conducted a qualitative screening and GC‐MS characterization of aqueous extracts of green and cured *Nicotiana tabacum* leaves and detected the presence of several secondary metabolites such as alkaloids, phenolics, flavonoids, tannins, terpenoids, saponins, and steroids [26]. Prommaban et al. have done the same to evaluate the quantities of carbohydrates, glycosides, terpenoids, steroids, alkaloids, tannins, and anthraquinones in two different varieties of *N. tabacum* (*N. tabacum* var. Virginia and Turkish) while using two types of extracts: ethanolic extract and petroleum ether extract [27]. The results have shown that terpenoids, steroids, alkaloids, and tannins were found in all extracts, but only carbohydrates were found in the ethanolic extracts of both varieties. In contrast, glycosides and anthraquinones were totally absent.

Oeung and Yin tested different extracts of *N. tabacum* leaves: aqueous extract, methanol extract, ethanol extract, ethyl acetate extract, and chloroform extract [28]. The aqueous extract of *N*. *tabacum* leaves contained alkaloids, tannins, flavonoids, steroids, cardiac glycosides, essential oils, resins, and polypeptides; its methanol extract expressed alkaloids, phenolic compounds, steroids, terpenoids, cardiac glycosides, essential oils, resins, saponins, and quinones. The ethanol extract of the substance was tested and found to contain alkaloids, phenolic compounds, flavonoids, steroids, terpenoids, essential oils, resins, saponins, quinones, and polypeptides. The ethyl acetate extract revealed the presence of tannins, terpenoids, saponins, quinones, and polypeptides. Similarly, the chloroform extract contained alkaloids, steroids, terpenoids, cardiac glycosides, essential oils, quinones, and polypeptides.

On the other side, Sharma et al. have studied the phytochemical composition of the stems of *N. tabacum*, and the results have shown that saponins, flavonoids, terpenoids, and alkaloids were found to be present in the aqueous extract. The ethanolic and methanolic extracts comprise saponins, flavonoids, and alkaloids only [29]. This study reveals that the stems have significantly fewer components than the leaves.

### 3.1. Alkaloids

Alkaloids are nitrogen‐containing molecules with a low molecular weight. The presence of nitrogen atom in the alkaloid structure gives the compound drug properties [30]. They are known for their strong biological activity and are thought to serve as plant defense chemicals [31]. The presence of alkaloids, such as nicotine, nornicotine, anabasine, and anatabine, is a characteristic of *Nicotiana* species. Nicotine is the major alkaloid that accumulates in the leaves of most *N. tabacum* varieties, forming 90%–95% of the total alkaloid content [32]. These alkaloids are produced in the roots and carried via the xylem stream to the leaves, where they accumulate [25].

**FIGURE 2:**
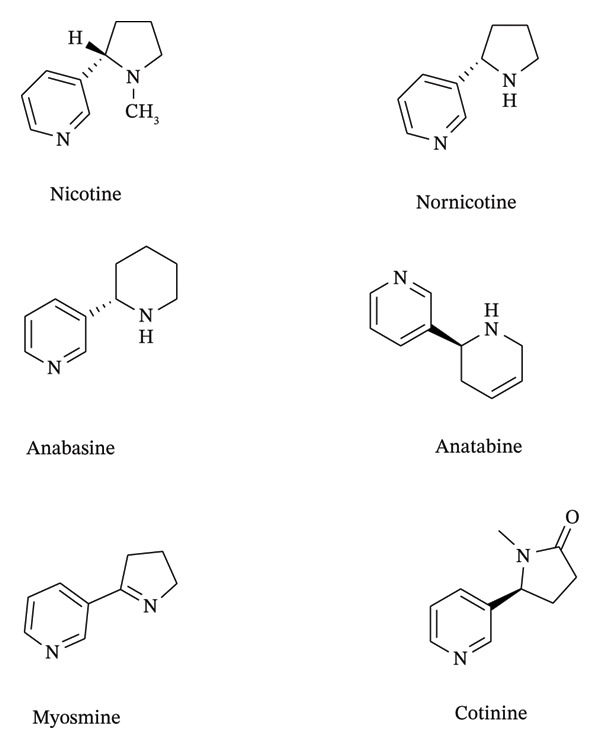
Structures of *Nicotiana tabacum* alkaloids.

Sun et al. identified six alkaloids from five types of commercial tobacco (one of them is *Nicotiana tomentosiformis*) via GC‐MS analysis: nicotine, nornicotine, myosmine, anabasine, anatabine, and cotinine (Figure 2) [33]. Nicotine was the predominant one followed by anatabine, nornicotine, and anabasine. Nornicotine was the abundant alkaloid in *N*. *tomentosiformis*.

Nicotine 3‐(1‐methyl‐2‐pyrrolidinyl) pyridine (C_10_H_14_N_2_) synthesis starts from a molecule named putrescine which could be synthesized through two routes; direct route from ornithine by ornithine decarboxylase (OCD) or indirectly from arginine by arginine decarboxylase (ADC). It is then hydrolyzed to N‐carbamoyl putrescine by agmatine iminohydrolase and then to putrescine by N‐carbamoyl putrescine amidohydrolase. Then, putrescine is converted into different forms to finally form the N‐methyl‐Δ‐pyrrolinium cation that contains the pyrrolidine ring [28, 30]. The pyridine ring of nicotine is formed from nicotinic acid [34]. Nicotinic acid in plants is derived from quinolinate [35]. Nicotine is formed by a condensation reaction of nicotinic acid and N‐methyl‐Δ‐pyrrolinium cation. This alkaloid is further metabolized to various alkaloids such as N‐formyl‐nornicotine, nornicotine, myosmine, nicotyrine, and cotinine [35]. As mentioned before alkaloids are synthesized in roots and then transported. Special proteins known as transporters help transfer nicotine all through the plant. Some of these transporters carry nicotine to the leaves (jasmonate‐inducible alkaloid transporters (JAT1 and JAT2)), which are stored in particular compartments known as vacuoles. Others transfer nicotine to the roots by multidrug and toxic compound extrusion (MATE 1 and MATE 2). A protein called nicotine uptake permease 1 (NUP 1) also helps transfer nicotine from the soil to the roots [31].

Aside from its defensive function, nicotine is responsible for the majority of the addictive traits of tobacco. It binds stereoselectively to nicotinic cholinergic receptors (nicotinic acetylcholine receptors (nAChRs)) and exerts its psychoactive effects by inducing pleasure, reducing anxiety and stress, and enhancing focus [35–37]. nAChRs are pentameric transmembrane cation channels that belong to ligand‐gated cation channels widespread throughout the nervous system and body [38]. They can be separated into two main categories: neuronal and muscle receptors. They are located on axon terminals, axons, dendrites, and somata in almost every area of the brain, both pre‐ and post‐synaptically [39]. Nicotine stimulates central nAChRs, leading to the release of many neurotransmitters, including dopamine. Furthermore, nicotine causes the release of other neurotransmitters such as norepinephrine, acetylcholine, serotonin, GABA, glutamate, and endorphins, which impact the many behaviors linked with nicotine use [36]. It has even been used therapeutically to reduce fatigue and exhaustion, purge nasal passages, or treat syphilis [40].

Like many alkaloids, nicotine is known for its toxicity. Textbooks, databases, and safety sheets agree that the lethal dosage for adults normally falls between 30 and 60 mg; the 60 mg dose corresponds to an oral LD_50_ of approximately 0.8 mg/kg [41]. Moderate acute toxicity symptoms may include nausea and vomiting. With continued exposure, these symptoms may progress to cholinergic syndrome, which includes diarrhea, increased salivation and respiratory secretions, and bradycardia. In extreme circumstances, poisoning can lead to seizures and respiratory depression [42].

### 3.2. Phenolic Compounds

Phenolic compounds are small molecules that contain at least one phenol unit in their structure [43]. Phenol is a benzene ring substituted with a hydroxyl group. Its systematic name is hydroxybenzene [44]. Plants produce several thousand of these compounds, which help to protect against UV‐B radiation, act as important pigments for the coloring of fruits and flowers, provide defense against herbivores, and prevent the penetration of microorganisms into the plant [8]. Phenolic compounds can be categorized using a variety of criteria. A frequent way is to pigeonhole them according to the number of aromatic rings, followed by their carbon skeletons and finally by their basic chemical structures [45]. They could be simple phenolic compounds such as simple phenolics and phenolic acids, polyphenols, flavonoids, and tannins. There are also other phenolic compounds like stilbenes, ligans, and lignins [43, 45]. Several research studies were conducted on tobacco plants to identify their phenolic compounds. Chen et al., 2012, showed that *Nicotiana* leaves contain several types of phenolics: chlorogenic acid, 3‐O‐caffeoylquinic acid, 5‐O‐caffeoylquinic acid, quercetin, rutin, kaempferol 3‐O‐rutinoside, esculetin, scopoletin, scopoline, p‐coumaric acid, caffeic acid, ferulic acid, (+)‐1‐hydroxy‐1,3,5‐ bisabolatrien‐10‐one, and 4‐ hydroxy‐3‐methoxybenzoic acid. During their research, they discovered additional compounds known as nicotphenols A, B, and C [46].

### 3.3. Terpenes

Terpenes are a large group of phytochemical compounds, with over 23,000 known structures. These compounds serve different functions in plants, such as being hormones (e.g., gibberellins and abscisic acid), photosynthetic pigments (e.g., phytol and carotenoids), electron transporters (e.g., ubiquinone and plastoquinone), and structural components of membranes (e.g., phytosterols). Terpenoids are generally colorless and fragrant, and they are insoluble in water but soluble in organic solvents. They are mainly found in the form of volatile oils, known as essential oils, in medicinal and aromatic plants [46–48]. Terpenes are polymeric compounds derived from isoprene (C_5_H_8_)n, classified by the number of isoprene units they contain. There are hemiterpenes with *n* = 1 (C5), monoterpenes with *n* = 2 (C10), sesquiterpenes with *n* = 3 (C15), diterpenes with *n* = 4 (C20), sesterterpenes with *n* = 5 (C25), triterpenes with *n* = 6 (C30), tetraterpenes with *n* = 8 (C40), and polyterpenes with *n* > 40. Terpenoids are also present in the form of various oxygenated derivatives, including alcohols, aldehydes, carboxylic acids, ketones, esters, and glycosides [48–51].

Many classes of terpenoids were detected in *N*. *tabacum*. Putri et al. and Khan et al.’s studies have elucidated that *Nicotiana’s* leaf extract contains triterpenoids [52, 53]. There are also sesquiterpenes, such as dehydrololiolide and 3‐oxo‐actinidol, along with nicosesquiterpenes, tabsesquiterpenes, norsesquiterpenes, and so on [51–54]. It also contains cembranoids, solanesol, and many other compounds [55, 56]. Tobacco is the only plant that accumulates large amounts of solanesol, a polyisoprenoid alcohol. This substance has potential applications as medicine, coenzyme Q10, and vitamin K2. [57].

### 3.4. Glycosides

Glycosides are a diverse class of secondary metabolites, consisting of a saccharide (mono‐ or oligosaccharide) or uronic acid linked via a glycosidic bond to a noncarbohydrate chemical compound called an aglycone. The main groups of glycosides include cardiotonic glycosides, cyanogenic glycosides, glucosinolates, and saponins [58].

Previously we mentioned that *N. tabacum* contains saponins and cardiac glycosides. The name saponins is derived from the Latin “sapo” meaning soap because they have surface‐active properties that form soapy foam when mixed with water. They are amphiphilic glycoside compounds consisting of a hydrophilic part called a glycoside and a hydrophobic part called aglycone or sapogenin. Plants for self‐defense synthesize these metabolites; they act as allelopathic agents and help fight insects and pathogens. According to the aglycone structure, saponins are mainly divided into triterpene saponins, steroidal saponins, and steroidal glycoalkaloids [59, 60].

Cardiac glycosides are highly toxic, found in many plants, and typically phytochemicals consisting of an aglycon bound to one or more sugar molecules [61]. These secondary metabolites are known for their effects on the heart and myocardial contractility by increasing contractility through inhibiting the sodium–potassium ATPase pump [62].

## 4. Traditional Uses and Pharmacological Effects of *Nicotiana tabacum*

The native people of America used tobacco plant in raw, unprocessed, or cured forms for various purposes [63]. The book “Tobacco and Kentucky”, written by Axton, provides a comprehensive description and details the diverse applications of the plant [64]. Tobacco has been used for spiritual and hallucination purposes as well as for medicinal ones. In the past, the smoke of tobacco was used to cure colic, gastric, and bronchial difficulties. The leaves of tobacco were chewed to aid toothache and also used as toothpaste. Additionally, chewed tobacco was employed as a disinfectant for cuts and bruises, as an emollient for venomous bites, and to treat internal infections and inflammations. The plant was also taken internally as a purgative and cure for worms. Furthermore, the indigenous people of America used tobacco against body lice. The plant has other medicinal purposes including antidiarrheal, anesthetic, pain relief, treating catarrh, and healing wounds and burns [65].

Recent studies have provided scientific evidence for the plant’s historical therapeutic properties, which support its traditional usage. This review highlights some of these properties.

### 4.1. Antioxidant Effect

Four extracts (aqueous, methanol, hexane, and acetone) of *N.* tabacum fresh roots that were shade dried were studied by Al‐Lahham et al. to evaluate the antioxidant activity of the plant [66]. The antioxidant activity was tested employing the 2,2‐diphenyl‐1‐picryl‐hydrazyl‐hydrate (DPPH) method. The results have shown that hexane, acetone, and methanol extracts exhibited a potent and significant antioxidant activity with IC_50_ values of 2, 6, and 21 μg/mL, while Trolox used as a standard has an IC_50_ value of 2 μg/mL.

Ru et al.’s work evaluated the antioxidant properties of flavonoids and polysaccharides extracted from fresh leaves dried in an oven using DPPH, 2,2′‐azino‐bis(3‐ethylbenzthiazoline‐6‐sulphonic acid) (ABTS) and reducing power tests. The research revealed that flavonoids extracted from the plant exhibited a more potent antioxidant activity compared to polysaccharides which indicates that tobacco leaves could serve as a natural reservoir of antioxidants [67].

Different extracts of different varieties (Virginia and Turkish) of leaves were tested using the DPPH, ABTS, ferric reducing antioxidant power (FRAP), and ferric thiocyanate assay. The results were the same as those of the previous ones [27].

These studies confirm that *N. tabacum* extracts possess antioxidant potential, operating through different mechanisms.

### 4.2. Antimicrobial Effect

#### 4.2.1. Antibacterial and Antifungal Effects

Plenty of in vitro studies affirmed that extracts of different parts of tobacco plant have antibacterial activity against Gram‐positive and Gram‐negative bacteria. Sharma et al. affirmed that the different stem extracts have an antibacterial activity on *Bacillus amyloliquefaciens* and *Staphylococcus aureus* (Gram +), as well as *Escherichia coli* and *Pseudomonas aeruginosa* (Gram −) [29]. The maximum antibacterial activity was signed by the methanolic and ethanolic extracts against *Staphylococcus aureus.* The extracts of *N. tabacum* leaves showed the same result [67, 68]. Dai et al. extracted two new sesquiterpenes (1‐isopropyl‐5‐methoxy‐3,7‐dimethylnaphthalene and 3‐hydroxymethyl‐1‐isopropyl‐5‐methoxy‐7‐methylnaphthalene) and tested their antimicrobial activity against methicillin‐resistant *Staphylococcus aureus* (MRSA), a type of bacteria known for its antibiotic resistance. The following compounds have exhibited promising results in inhibiting the growth of MRSA [69].

Khan et al. conducted several in vitro and in silico studies to evaluate the antimicrobial activity of the plant from different varieties against four bacterial strains (*S. typhi*, *E. coli*, *B. subtilis*, and *S. aureus*) and four fungal strains (*T. harzianum*, *A. brasiliensis*, *C. albicans*, and *A. niger*) [54]. The in vitro tests were evaluated by calculating the zone of inhibition and the urease inhibition. All the extracts from the four different varieties of *N. tabacum* possessed significant antibacterial and antifungal activities against the experimented strains. Additionally, the extracts expressed their capacity to inhibit the urease enzyme. This test was followed by a computational study to investigate the binding mode of two highly representative components of the polyphenolic fraction of *N. tabacum* leaves, namely, rutin and chlorogenic acid with urease. Docking has shown that these phenolic compounds may interact with the active site of urease.

#### 4.2.2. Antiviral Effect

The secondary metabolites of *N. tabacum* have a potent antiviral activity; the three flavones (tabaflavones A, B, and C), the sesquiterpenes (noreudesmanes 1, 2, and 3 and tabasesquiterpenes A–C), and the tabaisocoumarins (A–C) exhibited their antitobacco mosaic virus (anti‐TMV) activities [69–72]. Shedding light on denture hygiene, Kristiana et al. revealed that tobacco leaves can be utilized as a base for effervescent tablets as a denture cleaner due to its effective inhibition of *C. albicans* [73].

### 4.3. Antiparasitic Effect

Bahmani et al. confirmed that *N*. *tabacum* represents an antiparasitic activity after treating the leaches with different extract concentrations for 30 min [74]. The data showed that the LD_50_ values for *N. tabacum* were 13 × 10^4^ ppm which was considerable compared with the positive control (copper sulfate and NH_4_Cl). Tobacco was also studied to assume the effectiveness of its extract on immature and adult stages of *Rhipicephalus (Boophilus) microplus* ticks. The study found 100% mortality in nymphal and adult ticks at 36 h post‐treatment with 60% concentration and at 48 h post‐treatment with concentrations of 15%, 30%, 45%, and 60%. Anshu et al. could confirm from this study that *N*. *tabacum* shows promise as an effective biocontrol agent for tick control [75]. Other pests controlled by *N. tabacum* include *Grapholita molesta (Busck)*, *Anopheles gambiae, and Culex* sp. [75–77].

### 4.4. Antidiabetic Effect

Kazeem et al. investigated the impact of various extracts with different concentrations of *N. tabacum* on inhibiting α‐amylase from *Aspergillus oryzae* and α‐glucosidase from *Saccharomyces cerevisiae* [78]. These enzymes are involved in carbohydrate metabolism and their inhibition can help manage diabetes. The results have demonstrated that aqueous plant extract at high concentrations expresses a high percentage of inhibition of α‐amylase with a noncompetitive mode. Meanwhile, the acetone extract exhibited a competitive inhibition of α‐glucosidase.

### 4.5. Cytotoxic Effect

Numerous studies have shown that *N. tabacum* could have cytotoxic activity. Three new biphenyls derivatives, tababiphenyls G, H, and I, isolated from the leaves of *N. tabacum* were evaluated by M. Zhou et al. for their cytotoxicity against several human cancer cell lines including NB4 (human leukemia cells), A549 (human lung carcinoma cells), SHSY5Y (human neuroblastoma cells), PC3 (human prostate cancer cells), and MCF7 (human breast adenocarcinoma cells). The isolated biphenyl compounds demonstrated moderate cytotoxic activities against these human tumor cell lines, with IC50 values ranging from 2.8 to 9.4 μM [79].

In comparison, Chien et al. extracted different compounds from tobacco leaves, including 8‐formyl‐4′‐hydroxy‐6,7‐dimethoxyflavone, quercetin, (2R,3R)‐3,5‐dihydroxy‐7‐methoxyflavanone, kaempferol, and rutin. The two flavones showed a potent cytotoxic effect against cancerous cell lines, 8‐formyl‐7‐hydroxy‐6,4′‐dimethoxyflavone against CCRF‐CEM (human lymphoblastic leukemia) and P388D1 (murine macrophage‐like lymphoma) cell lines, with IC50 values of 2.24 ± 0.32 μg/mL. Meanwhile, 8‐formyl‐4′‐hydroxy‐6,7‐dimethoxyflavone exhibited cytotoxicities with IC_50_ values of 3.40 ± 0.21 μg/mL against the same cell lines [80]. Since these findings are based on in vitro tests that might not accurately predict in vivo action, they should be interpreted with caution. Potency may also be impacted by variations in chemical purity and extraction techniques. Nevertheless, the information shows that *N. tabacum* is a potential source of cytotoxic metabolites that merits more preclinical research.

### 4.6. Other Effects

According to Vaziri et al., a decoction of *N. tabacum* leaves can be used as a mouthwash with antiaphthous activity [81]. The neuroprotective effect was confirmed by Yang et al. who isolated cembranoids from the leaves and tested them against oxygen–glucose deprivation (OGD)‐induced neurotoxicity in SH‐SY5Y cells, and one of these compounds exhibited a protective effect against the OGD‐induced neurotoxicity [82].

Even if an extract shows high bioactivity in vitro, this does not always indicate the same level of activity in the raw plant material because some populations have historically consumed raw or unprocessed tobacco leaves, which can cause a significant difference in their phytochemical profiles from processed extracts.

Several studies have shown that processing, especially curing, alters the chemical and biological properties of *N. tabacum* leaves compared to raw material. Chen et al. (2021) demonstrated that air‐, sun‐, and flue‐curing significantly reduce nicotine, nitrogen, starch, polyphenols, and pigments [83], while Zou et al. reported similar changes with drying processes [84]. Regassa and Chandravanshi found higher levels of heavy metals in processed leaves than in raw leaves. Moreover, they did toxicological comparisons between raw and cured leaves [85]. It was also confirmed that curing affects not only the chemical composition but also the biological activity [86]. Since commercial tobacco products are typically produced from cured leaves, these findings underscore the importance of distinguishing between raw and processed material in both phytochemical and toxicological assessments. These results highlight the significance of differentiating between raw and processed material in both phytochemical and toxicological evaluations, as commercial tobacco products are usually made from cured leaves [87].

## 5. Tobacco Products

Tobacco products can be categorized as either smoking tobacco (ST) or SLT. The primary difference between smoking and SLT is the method of consumption. ST is a type of practice that involves the burning of tobacco. This method is commonly done by rolling tobacco into cigarettes. Various forms of tobacco are smoked besides cigarettes, such as cigars, pipes, kreteks, bidis, and waterpipes [88]. SLT products are not burned or inhaled like traditional cigarettes or cigars. Instead, these products are available for oral and nasal uses.

### 5.1. SLT Products

Although SLT is widely used in certain regions of the world, especially in the Eastern Mediterranean Region. SLT does not receive the same amount of attention as cigarette smoking, that is why in this review we aimed to shed light on it.

**FIGURE 3:**
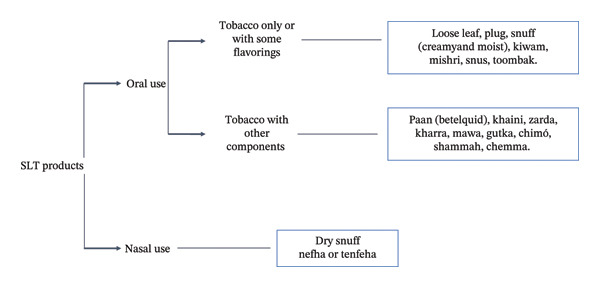
Different forms of SLT products.

As mentioned before, SLT products have different uses (oral and nasal uses); each mode of use contains numerous types of products [89]. Some products are commercially available, or users may fabricate what they want from ingredients. The oral products are meant to be chewed, sucked, and dipped. They are often placed in the space between the lower lip and gums or the space between the gums and the cheek. These products can be composed of tobacco leaves only as they can be a mixture of tobacco plant and other components. In this review, we will mention many known products such as snus, paan, gutka, khaini, zarda, loose leaf, kharra, kiwam, mishri, mawa, gudakhu, naswar, snuff (dry and moist), toombak, chimó, twist, shammah, chemma, and plug [90, 91]. Figure 3 resumes the previous information.

#### 5.1.1. Paan/Pan or Betel Quid With Tobacco

**FIGURE 4:**
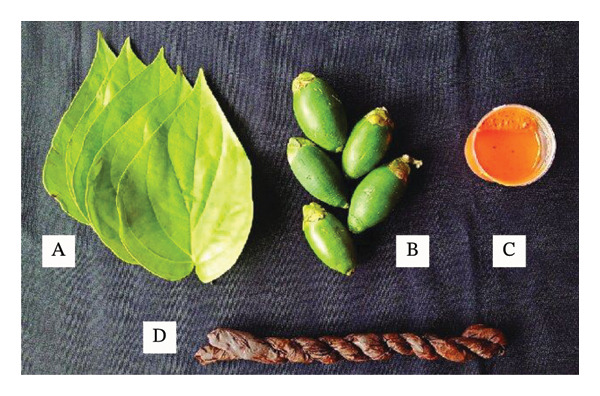
Components of betel quid: A, betel *Piper* vine leaf; B, areca nut; C, lime from ground and burnt sea shells; and D. tobacco [92].

Betel quid (paan or pan) is a type of SLT products that is sucked or chewed, made in India, and widely used throughout Asia. It is a combination of betel leaf (*Piper betle*), areca nut (*Areca catechu*), slaked lime (calcium hydroxide), and tobacco (Figure 4). They can contain other substances, particularly spices, including cardamom, saffron, cloves, aniseed, turmeric, mustard, or sweeteners [91, 93, 94].

Other SLT products, such as kharra and mawa, may contain areca nut or catechu [91].

#### 5.1.2. Khaini

Khaini is a product made by rubbing a pinch of coarsely cut, sun‐dried tobacco leaves together with slightly moistened slaked lime paste. Some users may add areca nut to it. The tobacco used for khaini is from *N. rustica* and/or *N. tabacum.* It is India’s most common SLT product. Khaini products are held in the mouth and sucked or chewed [95, 96].

#### 5.1.3. Zarda

In the UK, Zarda product is moist or dry chewing tobacco mixed with a variety of colorings, spice essences, and perfumes [97], while in India and southern Asia, it is a mixture of tobacco, lime, spices, and silver flakes [91]. According to Niaz et al., the ingredients of zarda include dried and boiled tobacco leaves, lime, and areca nut. Additives are also used, and it is commonly consumed in India and Arab countries [98].

#### 5.1.4. Loose Leaf, Twist, Plug, and Snuff

**FIGURE 5:**
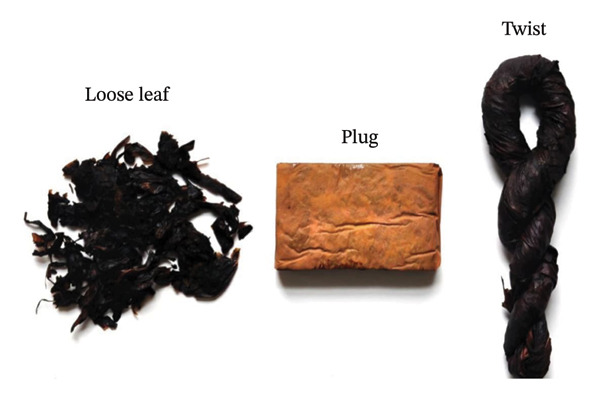
Different types of chewing tobacco adapted from [99].

**FIGURE 6:**
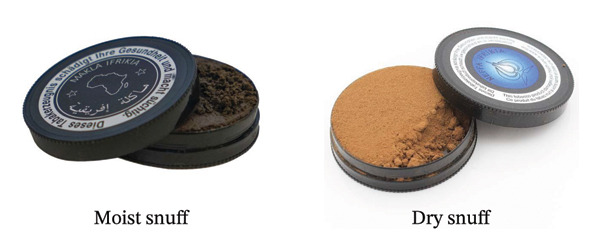
Moist and dry snuff adapted from [100].

Four primary types of SLT products are commonly used in the United States: loose leaf, twist, plug (Figure 5), and snuff (Figure 6). Loose‐leaf chewing tobacco usually comprises air‐cured tobacco and is flavored with licorice and sweeteners. Meanwhile, plug tobacco is made from the denser leaves taken from the plant’s top. These leaves are separated from their stems, soaked in a mixture of licorice and sugar, compressed into a plug, rolled in a wrapper leaf, and reshaped. Twist tobacco is formed from cured tobacco, air‐cured and fire‐cured leaves. These leaves are flavored and twisted to form a rope [96].

**FIGURE 7:**
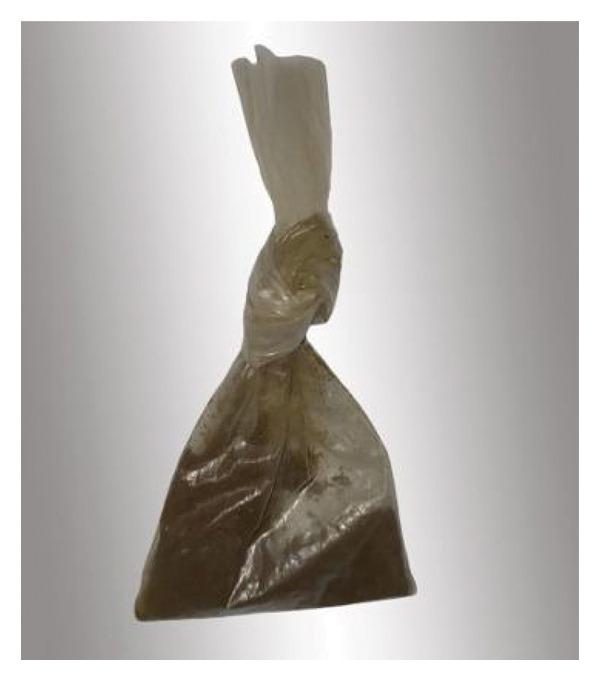
Sachet containing tenfeha/nefha product (taken and edited by Abir Chami).

The indigenous people of America also used snuff which was a fine aromatic powder composed of dried and thin leaves combined with tobacco, roots, peels, and seeds. However, commercial snuff is made solely from industrialized tobacco and does not contain other medicinal plants or serve any therapeutic or spiritual purposes [101]. Snuff has different forms, dry and moist (Figure 6). Dry snuff can be used orally and nasally and consists of fermented, finely ground fire‐cured tobacco [102]. In Morocco, dry snuff is commonly known as nefha or tenfeha (Figure 7), and it is typically sniffed. However, some consumers may also wrap a small amount in tissue and use it as a dip; it i’s called *El’Kalla*. Moist snuff (dip) is made from dark air and dark fire‐cured tobaccos with moisture content [102].

#### 5.1.5. Swedish Snus

**FIGURE 8:**
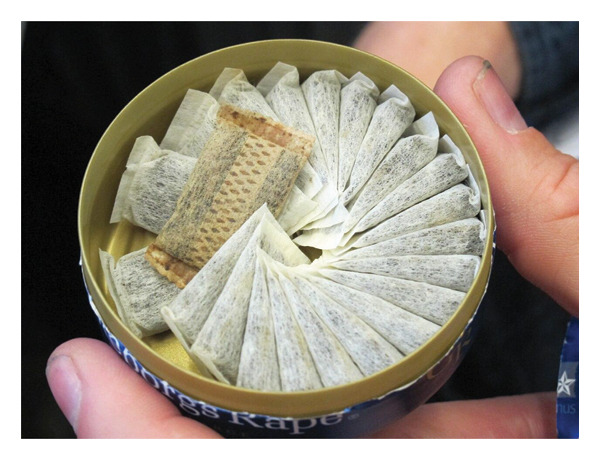
Snus product [103].

The Swedish snus (Figure 8) is a traditional Scandinavian tobacco snuff that is finely powdered and moistened. It is wrapped in small porous pouches and placed between the cheek and gum. When using snus, the saliva formed in the mouth is swallowed rather than spat out [104]. The processing of Swedish snus involves heat treatment or pasteurization, rather than the traditional fermentation of tobacco [9].

#### 5.1.6. Naswar

Naswar (Figure 9) is the most popular SLT product used by the population of the northwest areas of Pakistan, Afghanistan, Iran, and South Africa [106]. It is a mixture of sun‐dried tobacco leaves, slaked lime, wood ash, and flavorings. Sometimes coloring agents may be added to the mixture [107]. It is often held in the oral mucosa or occasionally under the tongue for approximately 30 min before spitting it out [108].

FIGURE 9Naswar product: (a) naswar powder and (b) naswar pouches [105].

**Figure:**
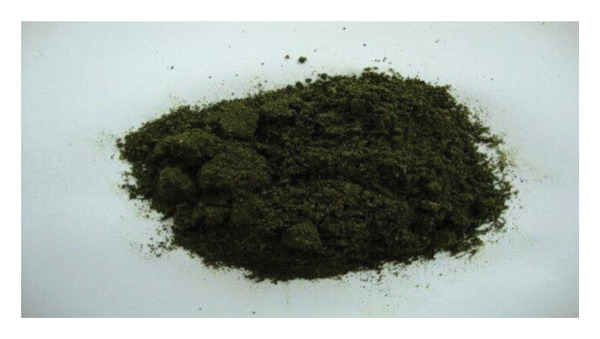
(a)

**Figure:**
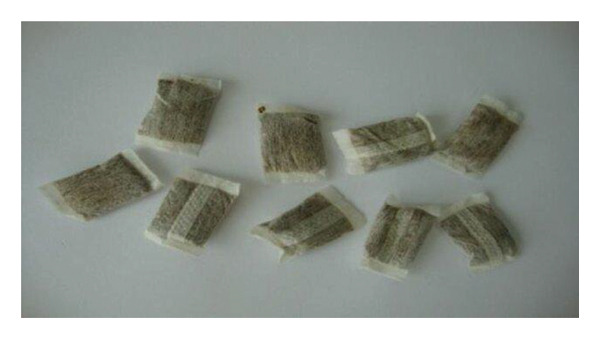
(b)

#### 5.1.7. Toombak

**FIGURE 10:**
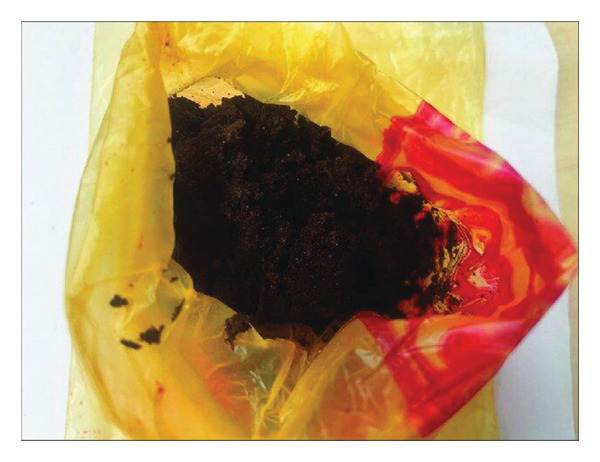
Toombak product [109].

Toombak (Figure 10) is a loose moist form of snuff that belongs to Sudan which was introduced about 400 years ago [110]. It is a type of SLT made by fermenting *N. rustica* leaves and adding sodium bicarbonate. It is commonly used as a dip, particularly by males [111].

#### 5.1.8. Shammah

**FIGURE 11:**
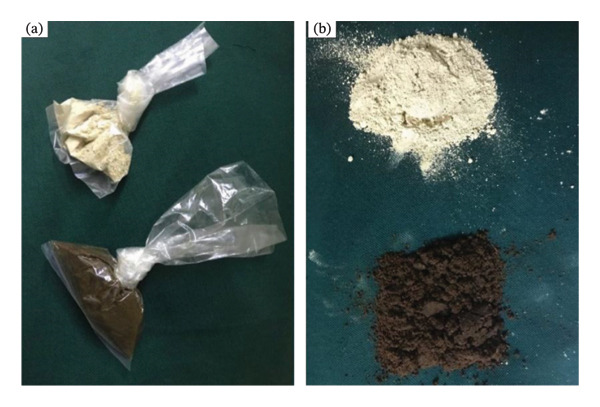
Shammah product: (a) packaged product and (b) loose product [112].

In the Arabian peninsula, shammah (Figure 11) is widespread, particularly in Saudi Arabia; it is a mixture of powdered tobacco, lime, ash, black pepper, oils, and flavorings. Like the previously mentioned products, shammah is placed in the buccal cavity of the mouth as well. The insoluble debris is spat out [113].

#### 5.1.9. Chemma

Chemma or chamma tobacco (CHT) (Figure 12) may be confused with shammah, but there is a difference in their composition, structure, and origin. No scientific data about this product are available; the following information is based on cultural knowledge and field observations. It is sometimes referred to as *El’Kalla* as well. It is a loose, moist, handmade product commonly used in the eastern (Oriental) region of Morocco; it originated in Algeria. It holds cultural significance and is enjoyed by many individuals in the region. It is a finely ground tobacco made from a blend of dried leaves and ashes (fig wood and juniper). The ash is used to give it that spicy taste. A few droplets of water are sprayed on the tobacco occasionally to create a green‐brown mixture that is moist to taste. Some spices may be added to the mixture. Some products may contain roots of the plant which are considered as low quality product. The mode of fabrication involves cutting the tobacco leaves using scissors. The leaves are then moistened by adding water to them. Ashes are added to the moistened leaves, and the mixture is thoroughly mixed and pressed to ensure proper blending of the ashes with the leaves. Finally, the prepared mixture is packed in either yellow or transparent small plastic bags (sachets) for consumption. It is taken by pinching the palate or placing it in the area between the lower lip and teeth. Some people could put it in different places as between fingers, toes, or down the armpits. CHT is widely used by males; it is rumored that its consumption helps to quit smoking cigarettes.

FIGURE 12(a) Chemma product in sachet. (b) Chemma product (taken and edited by Abir Chami).

**Figure:**
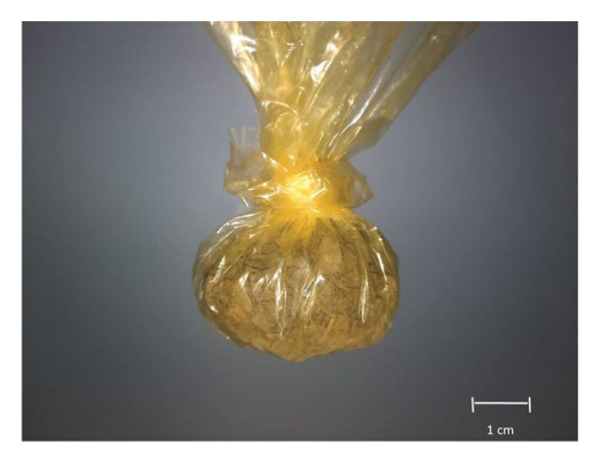
(a)

**Figure:**
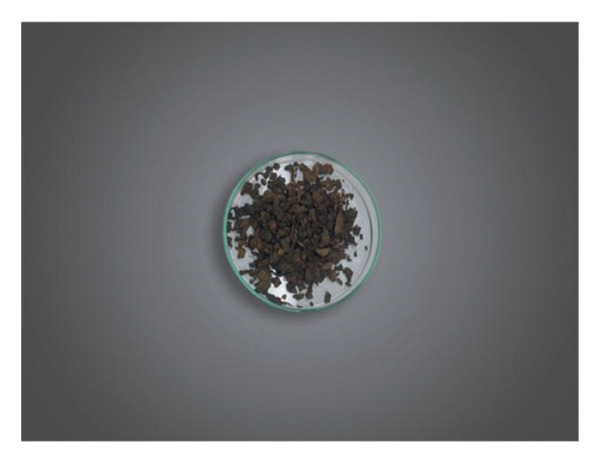
(b)

**FIGURE 13:**
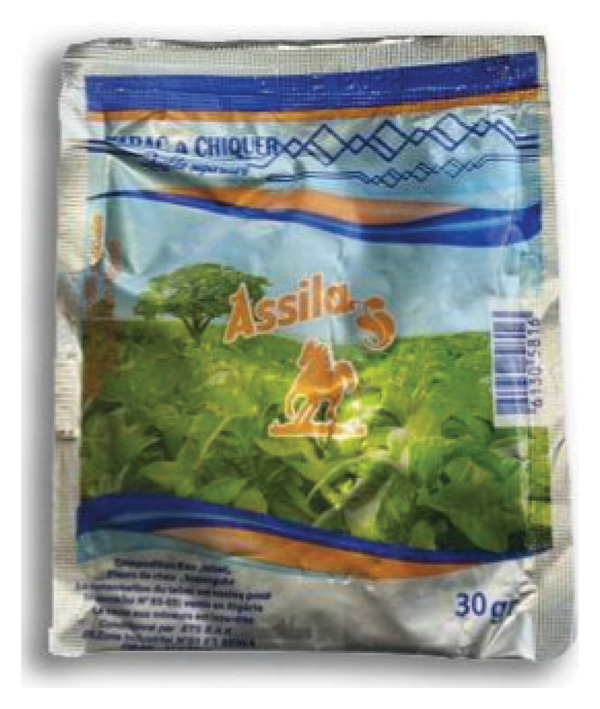
Picture of industrial SLT commercialized in the Oriental region of Morocco (taken and edited by Abir Chami).

Besides handmade CHT, there are other products (industrial) commercialized, but its utilization is not as prevalent as the traditional one*.* Many brands exist, but the well‐known one is Assila (Figure 13).

### 5.2. Side Effects of SLT Products

Many research studies were done on SLT products showing their harmful effects on human health. The first and the most obvious effect induced by SLT products is addiction; the main component inducing addiction is nicotine, which stimulates the brain’s reward system and causes desire for more tobacco use. As claimed by Henningfield et al. (1997), continued SLT use leads to tolerance, prompting users to increase nicotine intake by using more frequently or switching to products with higher nicotine concentrations [114]. The cessation of SLT consumption leads to withdrawal symptoms such as drowsiness, nervousness, headache, and irritability; some persons may have cravings [115]. Apart from nicotine, even though the chemical composition of SLT products varies depending on the composition, brands, and manufacturers, they all contain several common chemical components that can lead to dangerous effects. These include TSNAs, volatile N‐nitrosamines, N‐nitrosamino acids, polycyclic aromatic hydrocarbons (PAHs), radionucleotides, metals, metalloids, pesticide residues, humectants, aflatoxins, and mycotoxins [116]. At least 28 carcinogens are identified in SLT products [114, 115]; the most abundant ones are TSNAs formed through the nitrosation of tobacco alkaloids during the fermentation and curing phases [116–118]. Since most SLT products are used orally, the most marked effects are observed in the oral cavity. These effects include oral cancer, leukoplakia, erythroplakia, oral submucous fibrosis (if mixed with areca nut), loss of periodontal support (recession), and staining of teeth and composite restorations [119, 120]. Leukoplakia and erythroplakia are two precancerous lesions of the oral mucosa with a high potential for malignant transformation [121]. These lesions can be white or yellow‐brown and develop into worse. It has been shown that users of SLT are more likely to develop oral cancer in areas where the tobacco product is held, such as the buccal or alveolar surfaces [122]. In vivo studies on animal models such as rats and hamsters have shown that SLT products (shammah is one of these products) induce oral cancer [23, 122, 123]. Case studies confirm the association between SLT product consumption and head and neck cancers [121, 124]. A systemic review done by Gupta et al. informed that the major cause of tumor development in India is the continuous usage of SLT products (ghutka, zarda, mawa, kharra, khaini, and qiwam) [125]. Naswar users were found to have a risk of oral cancer more than 20 times higher than those who never used it [126]. In contrast, many researches have shown that the Swedish snus is not associated with oral cancer [125, 127]. During the manufacturing process, the product undergoes pasteurization, which eliminates TSNAs, explaining its lower levels of carcinogens [9]. Many SLT forms contain microorganisms; the consumption can cause damage to the oral layer and dysbiosis of oral microbiota and increase the risk of oral cancer [128]. We can also explain the link between SLT and oral cancer by how exposure to SLT can disrupt the normal anti‐inflammatory activity of annexin I while simultaneously increasing the production of pro‐inflammatory cyclooxygenase‐2 (COX‐2), which can lead to oral cancer [129]. SLT can increase the risk of having other types of cancers such as pancreatic and esophageal [128, 130].

Brinchmann et al. have mentioned in their systematic review that using the Swedish SLT (snus) during pregnancy is associated with several risks to pregnancy and newborn health, including stillbirths, premature births, reduced birth weight, and neonatal apnea, and a higher risk of oral cleft malformations was also observed in newborns [131]. Similar results were also found by Pratinidhi et al. [132]. Consuming SLT (mishri) during pregnancy is associated with adverse outcomes such as anemia; mishri users were found to be anemic (69.8%) compared to nonusers (16.3%). They also experienced higher rates of other complications like oligohydramnios, fetal distress, pregnancy‐induced hypertension, and past spontaneous abortion. Newborn babies of SLT users were noticed to have low birth weight and short height compared to nonusers.

Nicotine crosses the placenta which leads to significant placental damage, contributing to fetal growth and low birth weight. More than that it disrupts various reproductive processes by altering hormonal signaling and inducing oxidative stress [131, 133]. SLT products also affect male fertility; it was shown by Said et al. that tobacco chewing negatively impacts male fertility by impairing semen quality, disrupting testicular function, and increasing oxidative stress [134].

Consumption of SLT can cause other serious health consequences. Westman declared that SLT could lead to high blood pressure because of some ingredients such as nicotine sodium (varying from 0.61% to 3.53% depending on the type and brand) and licorice which are suspected of raising blood pressure [135]. Bolinder et al. have found that SLT users were more likely to have hypertension and other circulatory and respiratory diseases than nontobacco users or smokers [136]. Specifically, there was a noticeably higher risk of raised blood pressure among older SLT users (ages 45–56). SLT usage presents severe cardiovascular risks; smokers and SLT users were approximately 50% more likely to get disability pensions due to cardiovascular disease. Besides its effect on blood pressure, Choudhary and Qudeer affirmed from several tests that SLT users can have low lung function compared to nonusers [137].

Shaik et al.’s experiments have shown that SLT users (gutkha and khaini users) had significantly higher oxidative stress marker levels than nonusers [138]. These markers include plasma peroxynitrites, nitric oxide (NO), lipid peroxidation (LPO), and protein carbonyls (PCO). They indicate oxidative and nitrosative stress. The study also shown increased levels of total cholesterol, triglycerides, and very low‐density lipoprotein cholesterol (VLDL‐C), while the levels of high‐density lipoprotein cholesterol (HDL‐C) were significantly decreased. These markers indicate a higher risk of atherosclerosis and cardiovascular disease. Other indicators, such as increased creatinine and urea levels, showed significant alterations, indicating possible renal disease. There have also been reports of decreased hemoglobin and certain amino acid levels, which may indicate further systemic consequences of SLT.

Studies conducted by Zutshi et al. have found that some products can affect hemostasis and increase the risk of thrombosis. Specifically, oral SLT has been found to elevate fibrinogen and d‐dimer levels, as well as other inflammation markers in comparison to nontobacco users [139]. The elevation of fibrinogen contributes to hypercoagulability, increasing the risk of thrombosis. D‐Dimer is a product of cross‐linked fibrin degradation; high level of this product can indicate the presence of many diseases such as venous thromboembolism, pulmonary embolism, deep vein thrombosis, and other conditions like malignancies, inflammatory conditions, and infections [137, 138], which means the long‐term use can further increase the risk of atherothrombotic cardiovascular events among SLT users. Another study by Nicolas et al. described a situation in which a patient taking the anticoagulant drug (antivitamin K) did not achieve a therapeutic international normalized ratio (INR), a standardized measure used to assess the coagulation capacity of patients receiving anticoagulant therapy. Although the patient was taking high doses of antivitamin K, the patient’s INR remained below therapeutic levels. In this case, the patient admitted to daily use of SLT, specifically chewing tobacco, for 16 years. In this case, the authors suggest that the failure to achieve a therapeutic INR may be due to increased vitamin K exposure resulting from the patient’s chewing tobacco consumption. Indeed, tobacco contains high vitamin K levels, essential for normal blood clotting [140]. Increased vitamin K intake can counteract the anticoagulant effects of warfarin, leading to subtherapeutic INR levels.

It was shown that nicotine enhances platelet aggregation in response to agonists like adenosine diphosphate (ADP) and serotonin, both at higher and lower concentrations. It can also stimulate platelet activation and serotonin release, further enhancing platelet aggregation [141]. This explains the risk of thrombosis induced by nicotine‐containing products such as SLT ones.

Other works were also conducted on some SLT products. Malovichko et al. affirmed that nicotine, found in SLT products like “snuff” and “snus,” plays a role in the effects of chronic exposure. These products can decrease the levels of circulating endothelial progenitor cells (EPCs), endothelial microparticles, and immune cells. However, they also increase the levels of tumor necrosis factor‐alpha (TNF‐α) in the plasma [142]. EPCs are responsible for repairing damaged endothelial cells. A decrease in EPC levels can compromise the ability of the endothelium to repair itself, leading to impaired endothelial function and integrity [143]. As known the endothelium plays a critical role in maintaining vascular health, regulating blood flow, and preventing the formation of blood clots. Dysfunction of the endothelium can lead to the development of many issues.

Table 1 summarizes the health risks previously mentioned due to SLT consumption.

**TABLE 1:** Summary of SLT products, composition, and health effects.

SLT products	Origin	Composition	Health risks
Paan/pan	India	Betel leaf, areca nut, slaked lime, and tobacco Additives may be added	Cancer risks: oral, esophageal, lung, and cervical cancers [123, 125]. Oral/periodontal risks: leukoplakia, erythroplakia, erythroleukoplakia, oral submucous fibrosis, periodontal issues, and staining of teeth and dental restorations [123]. Other effects: addiction [115].

Khaini	India	Slaked lime paste and tobacco leaves Areca nut may be added	Cancer risks: oral, esophageal, lung, and prostate cancers [123, 125]. Oral/periodontal risks: leukoplakia, erythroplakia, erythroleukoplakia, oral submucous fibrosis, periodontal issues, and staining of teeth and dental restorations [123, 125, 144]. Other effects: addiction, inflammation, and thrombosis [115, 139].

Zarda	India and southern Asia	Tobacco, lime, spices, and silver flakes Additives may be added	Cancer risks: oral, esophageal, and lung cancers [123, 125, 144]. Oral/periodontal risks: leukoplakia, erythroplakia, erythroleukoplakia, oral submucous fibrosis, periodontal issues, and staining of teeth and dental restorations [123, 125, 144]. Other effects: addiction, inflammation, and thrombosis [115, 139]

Loose leaf, twist, plug, and snuff (dry and moist) or SLT products (generic, unspecified forms, or brands)	—	Tobacco and flavors (e.g., licorice and sugar)	Cancer risks: oral, head, and neck cancers [124, 144, 145]. Cardiovascular risks: hypertension, cardiopulmonary impairment, autonomic dysfunction, thrombosis risk, endothelial dysfunction, and inflammation [122, 135, 137, 139, 140]. Reproductive risks: placental damage and male infertility risk [134, 146]. ‐ Oral/Periodontal risks: leukoplakia, periodontal damage, and dental staining [122]. ‐ Other effects: addiction [115, 122].

Snus	Sweden	Tobacco snuff	Cancer risks: pancreatic cancer [125, 147]. Cardiovascular risks: hypertension, endothelial dysfunction, and inflammation [136, 139]. Other effects: addiction [115].

Naswar	Pakistan, Afghanistan, Iran, and South Africa	Tobacco, slaked lime, wood ash, flavorings, and colorings	Cancer risks: oral [123, 126, 144]. Oral/periodontal risks: leukoplakia, erythroplakia, erythroleukoplakia, oral submucous fibrosis, periodontal issues, and staining of teeth and dental restorations [123, 126, 144]. Other effects: addiction [115].

Toombak	Sudan	Tobacco and sodium bicarbonate	Cancer risks: oral cancer [123, 144]. Oral/periodontal risks: leukoplakia, erythroplakia, erythroleukoplakia, oral submucous fibrosis, periodontal issues, and staining of teeth and dental restorations [123, 144].

Shammah	Saudi Arabia	Tobacco, lime, ash, and flavorings	Cancer risks: oral cancer [123, 148]. Oral/Periodontal risks: leukoplakia, erythroplakia, erythroleukoplakia, oral submucous fibrosis, periodontal issues, and staining of teeth and dental restorations [123, 148]. Other effects: addiction [113].

Chemma/chamma	Eastern Region of Morocco	Tobacco and ash and flavorings may be added	No prior scientific investigations have been conducted.

Mishri	India	Burned tobacco	Cancer risks: oral cancer[123]. Oral/periodontal risks: leukoplakia, erythroplakia, erythroleukoplakia, oral submucous fibrosis, periodontal issues, and staining of teeth and dental restorations [123]. Reproductive risks: maternal anemia, preterm birth, and low birth weight [132]. Other effects: addiction [115].

Gutkha/gutka	India	Crushed areca nut, tobacco, slaked lime, catechu, and flavorings	Cancer risks: oral, esophageal, cervical, and prostate cancers [123, 125]. Oral/periodontal risks: leukoplakia, erythroplakia, erythroleukoplakia, oral submucous fibrosis, periodontal issues, and staining of teeth and dental restorations [123, 125]. Cardiovascular risks: cardiopulmonary impairment, autonomic dysfunction, oxidative and cardiometabolic stress, and thrombosis [137–139]. Other effects: addiction and inflammation [115, 139].

## 6. Conclusion

*Nicotiana tabacum*, a plant rich in phytochemicals, has interesting therapeutic potential. Historically, people used the plant for different therapeutic purposes to cure different diseases. Nowadays, these remedial effects have been proven by many scientific research studies. It was confirmed that *N. tabacum* has numerous pharmacological effects such as antioxidant, antimicrobial, antiparasitic, antidiabetic, cytotoxic, and many others. However, it is crucial to distinguish the plant itself from by‐products such as SLT products that are associated with various harmful effects on health, including an increased risk of cancer, particularly oral cancer, as well as heart and vascular disease and the risk of thrombosis.

The hemostatic studies on SLT products are scarce to prove the thrombotic effect of these products. More studies should be conducted to raise awareness about their detrimental effects.

**Nomenclature**
ABTS: 2,2′‐Azino‐bis(3‐ethylbenzthiazoline‐6‐sulphonic acid)
ADC: Arginine decarboxylase
Anti‐TMV: Antitobacco mosaic virus
CHT: Chemma tobacco
DPPH: 2,2‐Diphenyl‐1‐picryl‐hydrazyl‐hydrate
EPC: Endothelial progenitor cells
FRAP: Ferric reducing antioxidant power
JAT1 and JAT2: Jasmonate‐inducible alkaloid transporters 1 and 2
IARC: International Agency for Research on Cancer
MATE 1 and 2: Multidrug and toxic compound extrusion 1 and 2
MRSA: Methicillin‐resistant *Staphylococcus aureus*
nAChRs: Nicotinic acetylcholine receptors
NCI: National Cancer Institute
NUP1: Nicotine uptake permease 1
OGD: Oxygen–glucose deprivation
PAH: Polycyclic aromatic hydrocarbons
SLT: Smokeless tobacco
ST: Smoking tobacco
TNF‐α: Tumor necrosis factor‐alpha
TSNAs: Tobacco‐specific nitrosamines
USDA: United States Department of Agriculture
WHO: World Health Organization

## Author Contributions

Comprehensive literature review, writing–original draft, visualization, and revision and editing the manuscript: Abir Chami.

Writing–review and editing: Afaf Allaoui.

Writing–review and editing, supervision, and validation: Hassane Mekhfi and Younes Zaid.

## Funding

No funding was received for this manuscript.

## Disclosure

All scientific content, interpretations, and conclusions were developed, critically reviewed, and validated by the authors, who take full responsibility for the accuracy and integrity of the manuscript.

## Conflicts of Interest

The authors declare no conflicts of interest.

## Data Availability

Data sharing is not applicable to this article as no datasets were generated or analyzed during the current study.
